# Endoscopic findings of *Helicobacter pylori* gastritis in children and young adults based on the Kyoto classification of gastritis and age‐associated changes

**DOI:** 10.1002/jgh3.12652

**Published:** 2021-08-27

**Authors:** Mariko Hojo, Akihito Nagahara, Takahiro Kudo, Tsutomu Takeda, Tamaki Ikuse, Kohei Matsumoto, Kumiko Ueda, Hiroya Ueyama, Kenshi Matsumoto, Daisuke Asaoka, Toshiaki Shimizu

**Affiliations:** ^1^ Department of Gastroenterology Juntendo University School of Medicine Tokyo Japan; ^2^ Department of Pediatrics Juntendo University Faculty of Medicine Tokyo Japan

**Keywords:** child, endoscopy, *Helicobacter pylori*, young adult

## Abstract

**Background and Aim:**

We aimed to evaluate endoscopic findings of *Helicobacter pylori* (*H. pylori*)‐positive children and young adults based on the Kyoto classification, and to examine if there are age‐associated changes in *H. pylori*‐positive gastritis.

**Methods:**

*H. pylori*‐positive patients under 40 years old who underwent upper gastrointestinal endoscopy from 1 April 2009 to 15 February 2017 were included. Subjects were classified into the Pediatric (<20 years) and Young adult groups (20–39 years). The patients' endoscopic photographs were retrospectively re‐evaluated based on the Kyoto classification. We compared endoscopic findings between the two groups and examined alterations in the findings of *H. pylori*‐associated gastritis in 5‐year age groups.

**Results:**

Forty‐three and 163 subjects were classified into the Pediatric and Young adult groups, respectively. In the Pediatric group, nodularity was seen in the majority (93.0%); other endoscopic findings in order of decreasing frequency included mucosal swelling (32.6%), spotty redness (25.6%), diffuse redness (18.6%), and atrophy (9.3%). In the Young adult group, endoscopic findings included atrophy (66.9%), mucosal swelling (49.7%), spotty redness (39.3%), nodularity (31.9%), and diffuse redness (30.3%). The difference in prevalence of nodularity or atrophy between the two groups reached statistical significance (*P* < 0.0001 each). The rate of nodularity decreased with increasing age in the Young adult group. The rate of atrophy was 33% in young adult patients aged 20–24, and the rate increased to >65% in patients over age 25.

**Conclusion:**

*H. pylori*‐positive children had endoscopic findings besides nodularity based on the Kyoto classification. The prevalence of atrophy increased in patients over age 20.

## Graphical abstract

*Helicobacter pylori*‐positive children had endoscopic findings besides nodularity based on the Kyoto classification. In the Young adult group, the prevalence of nodularity decreased with increasing age, and the prevalence of atrophy was over 65% in patients aged 25 years and over.

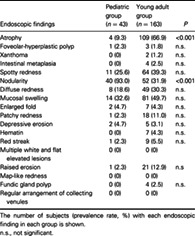

## Introduction

Schindler observed the living gastric mucosa through the gastroscope, and presented the features of chronic superficial gastritis, chronic atrophic gastritis, chronic hypertrophic gastritis, and lymphoblastomic gastritis in 1939.^1^ This was the first endoscopic classification of gastritis. Thereafter, *Helicobacter pylori* (*H. pylori*) was identified in 1982, and *H. pylori* has been established as a major cause of chronic gastritis. The Sydney system which ranks *H. pylori* as an important etiology of chronic gastritis, was proposed to classify chronic gastritis,^2^ and then the updated Sydney System^3^ was proposed. The system consists of a histological division and endoscopic division, and has been a commonly used classification for gastritis. An explanation and a photo of each endoscopic appearance have been published in papers on the Sydney system; however, objective definitions of edema and friability are obscure, and nodular gastritis which is a risk factor for gastric cancer^4^, ^5^ is not included in the category of gastritis. Then, the Kyoto classification of gastritis was proposed in 2013 to standardize useful endoscopic findings for diagnosing gastritis and a book on the Kyoto classification of gastritis was published.^6^ In this classification system, the following 18 endoscopic findings were defined: atrophy; foveolar‐hyperplastic polyp; xanthoma; intestinal metaplasia; spotty redness; nodularity; diffuse redness; mucosal swelling; enlarged fold; patchy redness; depressive erosion; hematin; red streak; multiple white and flat elevated lesions; raised erosion; map‐like redness; fundic gland polyp; and regular arrangement of collecting venules (RAC). In the Kyoto classification, pictures with a typical image of each of the 18 endoscopic findings are presented. The Kyoto classification is a kind of atlas. Physicians have judged endoscopic findings in each patient according to the pictures. The classification also showed relationships between these findings and the presence or absence of previously or present *H. pylori‐*infected gastric mucosa. The endoscopic finding of diffuse redness, mucosal swelling, or enlarged fold indicates *H. pylori*‐infected gastric mucosa; the endoscopic finding of map‐like redness indicates previously *H. pylori*‐infected gastric mucosa; and the endoscopic finding of RAC indicates *H. pylori*‐uninfected gastric mucosa.^7^ The endoscopic findings of atrophy, foveolar‐hyperplastic polyp, xanthoma, intestinal metaplasia, spotty redness, or nodularity do not indicate *H. pylori*‐uninfected gastric mucosa.

It is known that a typical endoscopic finding in *H. pylori*‐positive children is nodularity.^8^, ^9^, ^10^ However, it has been reported that nearly 60% and 15% of *H. pylori*‐positive children exhibited erythematous/exudative gastritis and atrophic gastritis based on the Sydney system, respectively.^11^, ^12^ Although various endoscopic findings were reported, these findings except for nodularity have not been fully described because standardized nomenclatures expressing endoscopic characteristics have not been developed.

We conducted the present study to evaluate the endoscopic findings of *H. pylori*‐positive children and young adults based on the Kyoto classification, to clarify the frequencies with which the endoscopic findings based on the Kyoto classification were observed, to compare the endoscopic findings between *H. pylori*‐positive children and young adults, and to study whether there are any age‐related changes in *H. pylori*‐positive gastritis.

## Material and methods

### Patients

We reviewed the endoscopic findings of *H. pylori*‐infected children and young adults in this retrospective study. Subjects were selected from patients who visited the department of gastroenterology or pediatrics at Juntendo University Hospital. Patients who underwent upper gastrointestinal endoscopy (UGI endoscopy) from 1 April 2009 to 15 February 2017, who were under 40 years old, and who were infected with *H. pylori* were included in this study. Patients were considered to be infected with *H. pylori* when at least one of the urea breath test with cutoff value of 2.5 per 1000 (Otsuka Pharmaceuticals, Tokyo, Japan), serum *H. pylori* antibody test with cutoff value of 10 U/mL (*E*‐plate; Eiken Chemical, Tokyo, Japan), and stool *H. pylori* antigen test (SRL, Tokyo, Japan) was positive. Patients in whom no endoscopic photographs had been taken were excluded. UGI endoscopy was performed using a video endoscopy system (Olympus GIF‐XQ260, GIF‐XP260, Q260, H260Z, or H290, Olympus, Tokyo, Japan).

### Study procedure and definition of endoscopic findings

Two experienced endoscopists who were board‐certified fellows of the Japan Gastroenterological Endoscopy Society (MH and TT) independently reviewed all photographs of endoscopic examinations during the study period retrospectively, and evaluated the endoscopic findings in the photographs based on the Kyoto classification, with disagreements resolved by consensus. Even in poor images, findings of the Kyoto classification were diagnosed within the visible range. Diffuse redness was defined as the presence of uniform redness involving the entire mucosa of the fundic gland. Mucosal swelling was defined as the presence of swelling of the gastric area in the fundic gland and/or thick, uneven gastric mucosa in the pyloric gland, and it was evaluated mainly by examining white light images. Atrophy was defined as visibility of vessels of variable size in the wall of the stomach and fold atrophy in the corpus area. The degree of endoscopic atrophy was classified into the closed type or open type according to the Kimura‐Takemoto classification.^13^ Further, the closed type is subdivided into C‐1, C‐2, and C‐3, and the open type is subdivided into O‐1, O‐2, and O‐3. Nodularity was defined as the presence of a small granular pattern resembling goose flesh in the gastric mucosa. Intestinal metaplasia was evaluated mainly by examining white light images. RAC was evaluated at the lesser curvature of the gastric angle and/or lower part of the corpus. The prevalence rate of each endoscopic finding was compared between the Pediatric group which consisted of patients less than 20 years old, and the Young adult group which consisted of patients who were 20 years old or over and under 40 years old. The changes in endoscopic findings in 5‐year age groups were assessed.

The study protocol was reviewed and approved by the Ethics Committee of Juntendo Hospital (#17‐190).

### Statistical analysis

This study was a retrospective study and was a pilot study without sample size calculation in which for the first time we compared endoscopic findings based on the Kyoto classification of gastritis between children and young adults. Fisher's exact test was used to compare the prevalence rate of endoscopic findings between the Pediatric and Young adult groups. A statistically significant level was defined as *P* < 0.05.

## Results

### Characteristics of the subjects

A total of 206 *H. pylori*‐positive patients fulfilled the study criteria. Forty‐three and 163 subjects were classified into the Pediatric and Young adult groups, respectively. The baseline characteristics of the patients in the two groups are summarized in Table 1. The most frequent reason for endoscopy in both groups was the presence of gastrointestinal symptoms such as abdominal pain, chest pain, heartburn, unusual sensation in the throat, nausea, or abdominal distension (81.4% in the Pediatric group and 44.2% in the Young adult group).

**Table 1:** Characteristics of the *Helicobacter pylori*‐infected patients in the two groups

	Pediatric group	Young adult group
	(*n* = 43; mean age ± SD, 11.7 ± 3.4 years)	(*n* = 163; mean age ± SD, 33.2 ± 4,8 years)
	*n*	*n*
Main reason for endoscopy		
Presence of gastrointestinal symptoms	35	72
Presence of *H. pylori*	2	23
Presence of bleeding or anemia	5	10
Precise examination	1	43
Screening	0	15
Age group (years)		
<5	1	
5–9	10	
10–14	25	
15–19	7	
20–24		6
25–29		35
30–34		36
35–39		86
Sex		
Male	22	68
Female	21	95

*H. pylori, Helicobacter pylori*; n, number of patients.

### Endoscopic findings of *H. pylori* gastritis based on the Kyoto classification of gastritis

**Figure 1:**
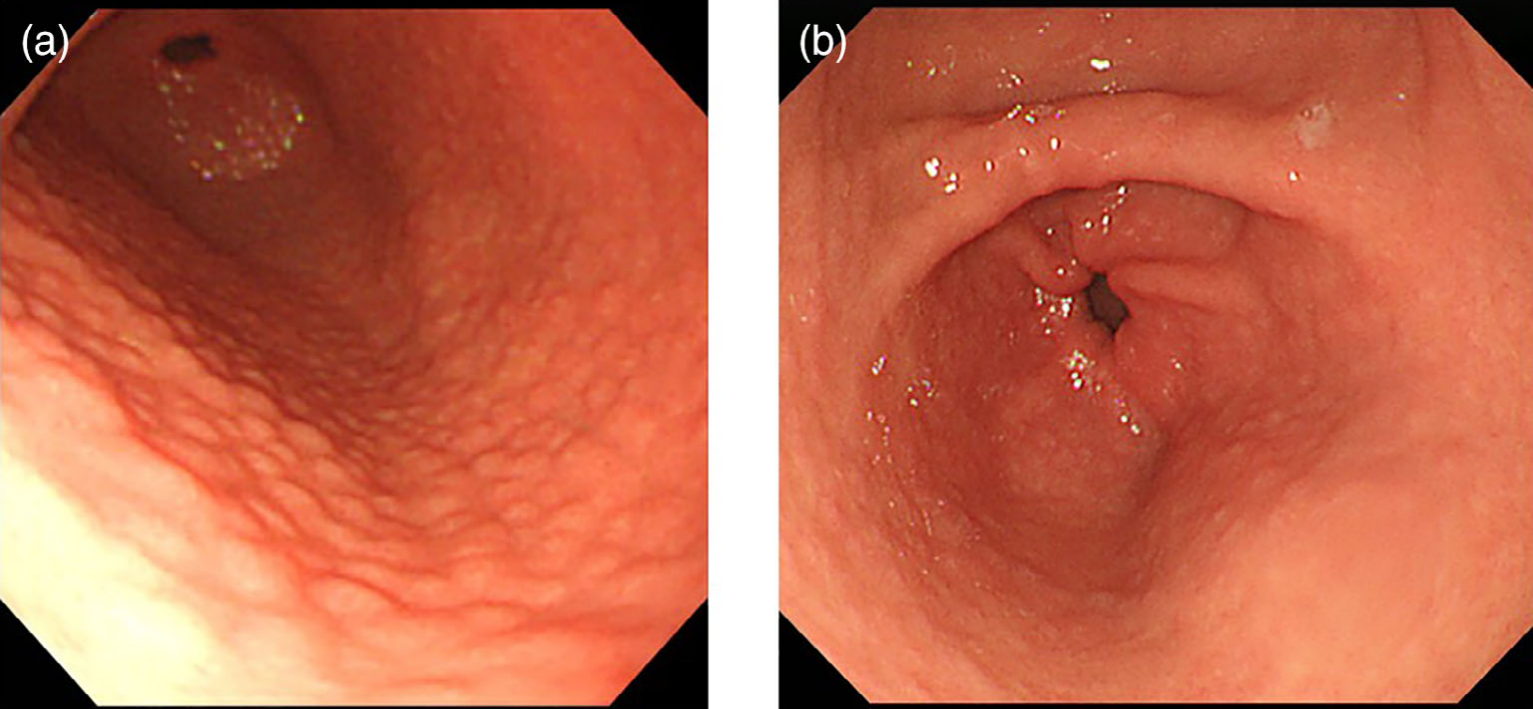
Endoscopic views of nodularity in *Helicobacter pylori*‐infected gastric mucosa. Endoscopic examination of *H. pylori*‐infected gastric mucosa showed a small granulated pattern resembling gooseflesh, which was classified as nodularity in the Kyoto classification of gastritis. (a) 13‐year‐old male. (b) 31‐year‐old female.

Comparisons of the numbers of subjects who had each endoscopic finding and the prevalence rate in the two groups are shown in Table 2. The endoscopic findings in order of decreasing frequency in the Pediatric group were nodularity (93.0%), mucosal swelling (32.6%), spotty redness (25.6%), diffuse redness (18.6%), atrophy (9.3%), enlarged fold (4.7%), depressive erosion (4.7%), foveolar‐hyperplastic polyp (2.3%), patchy redness (2.3%), red streak (2.3%), and raised erosion (2.3%). On the other hand, the endoscopic findings in order of decreasing frequency in the Young adult group were atrophy (66.9%), mucosal swelling (49.7%), spotty redness (39.3%), nodularity (31.9%), diffuse redness (30.3%), raised erosion (12.9%), patchy redness (11.0%), red streak (5.5%), enlarged fold (4.3%), hematin (4.3%), depressive erosion (3.1%), intestinal metaplasia (2.5%), fundic gland polyp (2.5%), foveolar‐hyperplastic polyp (1.8%), and xanthoma (1.2%). Nodularity (Fig. 1) was the most frequently observed endoscopic finding based on the Kyoto classification in the Pediatric group, and the prevalence rate of nodularity was significantly higher in the Pediatric group than in the Young adult group (93.0% *vs* 31.9%, *P* < 0.001). Nodularity was observed in 20 males (20/22, 91%) and 20 females (20/21, 95%) in the Pediatric group, while it was observed in 17 males (17/68, 25%) and 35 females (35/95, 37%) in the Young adult group. Atrophy was the most frequently observed finding in the Young adult group, and the prevalence rate of atrophy in the Young adult group was significantly higher than that in the Pediatric group (66.9% *vs* 9.3%, *P* < 0.001). Comparison of the prevalence rates of other endoscopic findings showed that significant differences between the two groups did not exist. The degree of atrophy was the closed type in all four subjects who had atrophy in the Pediatric group, and was the closed type in 88.1% (96/109) of the subjects who had atrophy in the Young adult group. Three subjects were classified as having C‐1 and 1 was classified as having C‐2 among the 4 subjects with atrophy in the Pediatric group, while 23 subjects were classified as having C‐1, 57 as having C‐2, 16 as having C‐3, 6 as having O‐1, 6 as having O‐2 and 1 as having O‐3 among the 109 subjects with atrophy in the Young adult group. Map‐like redness which has been observed in previously *H. pylori*‐infected mucosa was not observed in either group, and RAC which has been observed in *H. pylori*‐uninfected mucosa was also not observed in either group.

**Table 2:** Comparison of the prevalence of endoscopic findings between the pediatric and young adult groups

Endoscopic findings	Pediatric group (*n* = 43)	Young adult group (*n* = 163)	*P*
Atrophy	4 (9.3)	109 (66.9)	<0.001
Foveolar‐hyperplastic polyp	1 (2.3)	3 (1.8)	n.s.
Xanthoma	0 (0)	2 (1.2)	n.s.
Intestinal metaplasia	0 (0)	4 (2.5)	n.s.
Spotty redness	11 (25.6)	64 (39.3)	n.s.
Nodularity	40 (93.0)	52 (31.9)	<0.001
Diffuse redness	8 (18.6)	49 (30.3)	n.s.
Mucosal swelling	14 (32.6)	81 (49.7)	n.s.
Enlarged fold	2 (4.7)	7 (4.3)	n.s.
Patchy redness	1 (2.3)	18 (11.0)	n.s.
Depressive erosion	2 (4.7)	5 (3.1)	n.s.
Hematin	0 (0)	7 (4.3)	n.s.
Red streak	1 (2.3)	9 (5.5)	n.s.
Multiple white and flat elevated lesions	0 (0)	0 (0)	
Raised erosion	1 (2.3)	21 (12.9)	n.s
Map‐like redness	0 (0)	0 (0)	
Fundic gland polyp	0 (0)	4 (2.5)	n.s.
Regular arrangement of collecting venules	0 (0)	0 (0)	

The number of subjects (prevalence rate, %) with each endoscopic finding in each group is shown.

n.s., not significant.

### Age‐associated changes of endoscopic findings in *H. pylori*‐positive gastritis

The prevalence rate of each endoscopic finding in the patients with *H. pylori*‐associated gastritis in each 5‐year age group is shown in Table 3. The prevalence rate of nodularity was more than 95% in patients aged 10–19 years, and the rate decreased with increasing age in patients aged 20 years and over. The prevalence rate of atrophy was 33% in patients aged 20–24 years, and increased to >65% in patients aged 25 years and over. An age‐related trend was not observed in other endoscopic findings based on the Kyoto classification.

**Table 3:** Prevalence of endoscopic findings of *Helicobacter pylori*‐associated gastritis in each 5‐year age group

	Age group							
	<5 years (*n* = 1)	5–9 years (*n* = 10)	10–14 years (*n* = 25)	15–19 years (*n* = 7)	20–24 years (*n* = 6)	25–29 years (*n* = 35)	30–34 years (*n* = 36)	35–39 years (*n* = 86)
Atrophy	0 (0)	0 (0)	4 (16)	0 (0)	2 (33.3)	25 (71.4)	24 (66.7)	58 (67.4)
Foveolar‐hyperplastic polyp	0 (0)	0 (0)	0 (0)	1 (14.3)	0 (0)	1 (2.9)	0 (0)	2 (2.3)
Xanthoma	0 (0)	0 (0)	0 (0)	0 (0)	0 (0)	1 (2.9)	0 (0)	1 (1.2)
Intestinal metaplasia	0 (0)	0 (0)	0 (0)	0 (0)	0 (0)	2 (5.7)	0 (0)	2 (2.3)
Spotty redness	0 (0)	2 (20)	7 (28)	2 (28.6)	4 (66.7)	16 (45.7)	15 (41.7)	29 (33.7)
Nodularity	0 (0)	9 (90)	24 (96)	7 (100)	5 (83.3)	13 (37.1)	13 (36.1)	21 (24.4)
Diffuse redness	0 (0)	2 (20)	5 (20)	1 (14.3)	0 (0)	11 (31.4)	9 (25)	29 (33.7)
Mucosal swelling	0 (0)	2 (20)	10 (40)	2 (28.6)	1 (16.7)	20 (57.1)	14 (38.9)	47 (54.7)
Enlarged fold	0 (0)	1 (10)	0 (0)	1 (14.3)	0 (0)	2 (5.7)	0 (0)	5 (5.8)
Patchy redness	0 (0)	0 (0)	1 (4)	0 (0)	0 (0)	3 (8.6)	3 (8.3)	13 (15.1)
Depressive erosion	1 (100)	0 (0)	1 (4)	0 (0)	0 (0)	3 (8.6)	0 (0)	2 (2.3)
Hematin	0 (0)	0 (0)	0 (0)	0 (0)	1 (16.7)	3 (8.6)	1 (2.8)	2 (2.3)
Red streak	0 (0)	0 (0)	0 (0)	1 (14.3)	0 (0)	4 (11.4)	2 (5.6)	3 (3.5)
Multiple white and flat elevated lesions	0 (0)	0 (0)	0 (0)	0 (0)	0 (0)	0 (0)	0 (0)	0 (0)
Raised erosion	0 (0)	0 (0)	0 (0)	1 (14.3)	0 (0)	6 (17.1)	2 (5.6)	13 (15.1)
Map‐like redness	0 (0)	0 (0)	0 (0)	0 (0)	0 (0)	0 (0)	0 (0)	0 (0)
Fundic gland polyp	0 (0)	0 (0)	0 (0)	0 (0)	0 (0)	0 (0)	1 (2.8)	3 (3.5)
Regular arrangement of collecting venules	0 (0)	0 (0)	0 (0)	0 (0)	0 (0)	0 (0)	0 (0)	0 (0)

The number of subjects (prevalence rate, %) with each endoscopic finding in each group is shown.

## Discussion

Nodularity was observed in 93% of the *H. pylori*‐positive subjects in the Pediatric group, and was a typical endoscopic finding in the Pediatric group. Moreover, other endoscopic findings such as mucosal swelling, spotty redness, and diffuse redness were also observed in this group, and it had become clear that various endoscopic findings other than nodularity also existed in *H. pylori*‐positive children. Meanwhile, atrophy was observed in 66.9% (109/163) of the subjects in the Young adult group, among whom 88.1% (96/109) had the closed type of atrophy. Atrophy was the most frequently observed endoscopic finding in the Young adult group, while the prevalence rate of nodularity decreased to 31.9% in this group. It was reported that among *H. pylori*‐infected individuals, nodularity was observed more frequently in young adult females than in young adult males, although there was no gender difference in the presence of nodularity in children.^14^, ^15^ In our study, nodularity was also observed more frequently in females than in males in the Young adult group, while nodularity was observed in similar percentages of males and females in the Pediatric group. Map‐like redness and RAC were not observed in any of the subjects in our study. Map‐like redness which indicates previously *H. pylori*‐infected gastric mucosa and RAC which indicates *H. pylori*‐uninfected gastric mucosa were not observed in our *H. pylori*‐infected subjects regardless of their age. However, the detection rate of diffuse redness which indicates *H. pylori*‐infected gastric mucosa was low at 18.6% in the Pediatric group. The exact causes of this low rate are not clear. Diffuse redness may be a less common endoscopic finding in *H. pylori*‐infected Pediatric patients than in adult patients. It may have been the case that diffuse redness was not recognized because of poor images. Previously infected patients were included in the present study, and the diffuse redness in such subjects may have disappeared.

In our study we could clarify the natural course of endoscopic findings since acquisition of *H. pylori* infection up through young adulthood. The prevalence rate of nodularity was 93% in patients less than 20 years of age and the prevalence rate decreased with increasing age in patients over 20 years of age. On the other hand, regarding atrophy, the prevalence of atrophy was 9.3% in patients less than 20 years of age, 33% in patients aged 20–24 years, and >65% in patients over age 25 years. Features of endoscopic findings in the young adult *H. pylori*‐positive patients seemed to be an intermediate pattern between the features of endoscopic findings in adult patients and those in Pediatric patients.^16^, ^17^ Nodularity may regress by atrophic change with increasing age.^18^ Miyamoto *et al*. reported that the percentage of patients with nodularity in subjects aged more than 16 years who had undergone endoscopy was 0.19%, and they also reported that the prevalence of nodularity tended to decrease with increasing age.^14^ The prevalence rate of nodularity in patients aged 20 to less than 30 years in Miyamoto's study was 1.2%, while that in our study was 43.9% (18/41). The subjects in the study of Miyamoto *et al*. were patients who had undergone endoscopy regardless of *H. pylori* infection status and they diagnosed nodularity with endoscopic findings and pathologic features. On the other hand, our study subjects were patients who had undergone endoscopy and were infected with *H. pylori*, and we diagnosed nodularity endoscopically based on the Kyoto classification of gastritis. These differences could have resulted in the difference in the prevalence rates of nodularity. Akamatsu *et al*. reported that the endoscopic finding of atrophy was present in 60% of teenagers aged 16 and 17 years old.^19^ In the present study, atrophy was found in only 9.3% of the Pediatric group. In our study 84% of the subjects in the Pediatric group were under 15 years old, and the detection rate of findings was low in cases where the endoscopic images were poor. Such a difference and this limitation could have resulted in the difference in the prevalence rates of atrophy between the present study and the study of Akamatsu *et al*.^19^ Other endoscopic findings besides nodularity and atrophy did not show age‐related changes.

*H. pylori* progressively damages gastric mucosa, and severe atrophy and intestinal metaplasia are risk factors for gastric cancer in *H. pylori*‐infected patients.^20^ Because the prevalence rate of atrophy increased in the Young adult group, eradicating *H. pylori* at an age younger than 20 years may reduce the risk of gastric cancer. On the contrary, it was shown that there were patients in the Young adult group who still had nodular gastritis. Nodular gastritis in adults is considered to be a risk factor for gastric cancer.^4^, ^5^ *H. pylori*‐positive adults with nodular gastritis need to be carefully followed.

This study has several limitations. First, this study was a retrospective study. This study was a single‐center study, the sample size was limited, and the number of images taken by image‐enhanced endoscopy, such as linked color imaging which improves visibility,^21^ was small. In this study the prevalence rate of each endoscopic finding was investigated not in subjects of each age but in each 5‐year age group in order to clarify age‐associated changes, although an analysis according to real ages seems to be more useful. When *H. pylori* positivity is judged only by the antibody test, previously infected patients can be included.^22^ Accordingly, subjects who were not infected with *H. pylori* at the time of the endoscopic examination might have been included in this study. Because this study was a retrospective study, the endoscopic pictures were not taken by a standardized method, and many different types of scopes and endoscopic systems were used. Differences in the quality of endoscopic images caused by these factors may have led to differences in the sensitivity and specificity of the diagnosis. Actually, if particular endoscopic findings were not observed because of poor images, we judged that those endoscopic findings were absent. As a result, the detection rates of findings were likely lower than the actual rates. The reasons that endoscopy was performed in the included subjects were different in the two groups. Asymptomatic children rarely undergo endoscopy. In this study, the percentage of subjects without symptoms in the Pediatric group was lower than that in the Young adult group. Our results may have been affected by this selection bias.

In conclusion, the current study showed that *H. pylori*‐positive children had various endoscopic findings based on the Kyoto classification of gastritis besides nodularity. *H. pylori*‐positive children most frequently had the endoscopic finding of nodularity, followed by mucosal swelling, spotty redness, and diffuse redness. On the other hand, *H. pylori*‐positive young adults most frequently had atrophy, followed by mucosal swelling, spotty redness, nodularity, and diffuse redness. In the Young adult group, the prevalence of nodularity decreased with increasing age, and the prevalence of atrophy was over 65% in patients aged 25 years and over. In the future, it is necessary to carry out a prospective study with histological examination in order to verify the age‐related changes that were revealed by endoscopy in the present study.
